# Cytogenetic Findings of Patients with Acute Lymphoblastic Leukemia in Fars Province

**Published:** 2013-12

**Authors:** Akbar Safaei, Jahanbanoo Shahryari, Mohamad Reza Farzaneh, Narjes Tabibi, Marzieh Hosseini

**Affiliations:** Department of Pathology, School of Medicine, Shiraz University of Medical Sciences, Shiraz, Iran

**Keywords:** Acute lymphoblastic leukemia, Cytogenetic analysis, Chromosomal abnormalities, Incidence, Iran

## Abstract

**Background:** Acute lymphoblastic leukemia (ALL) is the sixth most common malignancy in Iran. Cytogenetic analysis of leukemic blasts plays an important role in classification and prognosis in ALL patients. The purpose of this study was to define the frequency of chromosomal abnormalities of ALL patients in adults and children in Fars province, Iran.

**Methods:** In this cross-sectional study, we evaluated karyotype results of bone marrow specimens in 168 Iranian patients with ALL (154 B-ALL and 14 T-ALL) in Fars Province using the conventional cytogenetic G-banding method.

**Results:** The frequency of cytogenetic abnormalities, including numerical and/or structural changes, was 61.7% and 53.8% in the B-ALL and T-ALL patients, respectively. Hyperdiploidy was the most common (32%) cytogenetic abnormality. Among structural abnormalities, the most common was t(9;22) in 11% of the patients. The children showed a higher incidence of hyperdiploidy and lower incidence of t(9;22) than adults (P<0.05). We found a lower incidence of recurrent abnormalities such as 11q23, t(1;19), and t(12;21) than those reported in previous studies.

**Conclusion: **Normal karyotype was more frequent in our study. The frequencies of some cytogenetic abnormalities such as hyperdiploidy and t(9;22) in our study were comparable to those reported in the literature. The results of this study in Fars Province can be used as baseline information for treatment decision and research purposes in ALL patients. We recommend the use of advanced molecular techniques in the future to better elucidate cryptic cytogenetic abnormalities.

## Introduction

Acute leukemia is a clonal expansion of white blood cell precursors in the blood, bone marrow, and various extramedullary tissues. The diagnosis of acute leukemia is based on the presence of more than 20% blasts in the peripheral blood or bone marrow. According to a recent study, hematologic malignancies are the sixth most commonly occurring malignancies in Iran in both sexes. Annual occurrence of leukemia disorders in the northwest of Iran was 3.7 (4.2–5.6) per 100,000 population.^1^

The French-American-British Cooperative classification of acute leukemia published in 1976, was based on morphology by the Romanowsky staining of bone marrow or peripheral blood films, and the World Health Organization (WHO) supervised classification according to molecular and cytogenetic studies.^2^ There are three varieties of recurrent genetic aberration in acute leukemia: (1) numerical abnormalities, including gain or loss of whole or segments of chromosomes; (2) balanced chromosomal translocations; and (3) molecular genetic abnormalities. Some of these abnormalities, including balanced translocation, have specific or diagnostic values, which may directly impact prognosis or therapeutic decision.^3^ The current WHO classification divides B-ALL into groups based on the frequently found cytogenetic findings. These groups are: (1) acute B lymphoblastic leukemia/lymphoma with hyperdiploidy; (2) acute B lymphoblastic leukemia/lymphoma with t(9;22)(q34;q11.2); BCR-ABL1; (3) acute B lymphoblastic leukemia/lymphoma with t(v;11q23); MLL rearranged; (4) acute B lymphoblastic leukemia/lymphoma with t(12;21)(p12;q22); TEL-AML1 (ETV6-RUNX1); (5) acute B-lymphoblastic leukemia/lymphoma with t(5;14)(q31;q32); IL3-IGH; (6) acute B-lymphoblastic leukemia/lymphoma with t(1;19)(q23;p13.3); E2A-PBX1 (TCF3-PBX1); (7) B lymphoblastic leukemia/lymphoma with hypodiploidy; and (8) acute B lymphoblastic leukemia/lymphoma not otherwise specified.^2^

Cytogenetic abnormalities in ALL are classified into three risk subgroups: (1) good; (2) intermediate; and (3) poor prognosis groups. High hyperdiploidy with 51–65 chromosomes and t(12;21)(p13;q22) is classified into the good-risk subgroup and is predominantly observed in children. Also, t(9;22)(q34;q11), a representative karyotype in the poor-risk subgroup, is primarily found in adults.^4^

The frequency of chromosomal abnormalities varies among populations, and this difference may be due to ethnicity and geographic factors.^5^ It is well known that cytogenetic data are vitally important in the diagnosis, treatment, and estimation of prognosis in ALL, especially in the pediatric group.^2^ Nonetheless, there are only a few reports from Iran on the frequency of leukemia karyotype abnormalities.^6^

In this study, we report cytogenetic findings on 168 cases of ALL patients in Fars province and compare the distribution of cytogenetic abnormalities between children and adults. The results from this study regarding the frequencies of cytogenetic abnormalities in ALL patients can be used for the classification of ALL according to the WHO groups, prognosis estimation, treatment decision, and research purposes.

## Materials and Methods

From March 2010 to August 2012, we reviewed all cases with a final diagnosis of ALL including 154 cases of B-cell type and 14 cases of T-cell type. Definite diagnosis in all the cases was established based on morphology, cytochemistry, immunohistochemistry, and flow cytometric analysis in our center. All the cases were referred from affiliated hospitals in Shiraz University of Medical Sciences. 

Pretreatment bone marrow aspirations or peripheral blood samples were cultured. Briefly, the samples were cultured in RPMI 1640 basal medium, containing 10% fetal calf serum (Gibco-Invitrogen-USA), for 72 hours at 37°C, and then treated with 0.1 microgram/ml of colcemid (Gibco-Invitrogen-USA) to stop the cells in the metaphase of mitosis. After harvesting with hypotonic solution (0.068 mol/L KCL) and fixation with acetic acid /methanol (1/3), the chromosomes were spread and stained using the standard G-banding technique. For each case, a minimum of 20 metaphases were analyzed by using the CytoVision® chromosomal karyotyping automatic system (Genetix Company-USA). Karyotype was written according to the International Chromosome Nomenclature (ISCN 2009). A successful cytogenetic analysis required the detection of at least 2 or more cells with the same structural change or chromosomal gain, 3 or more cells with the same chromosomal loss, in at least 20 metaphases.^7^ The patients’ karyotypes were thereafter subdivided into groups based on the WHO classification (2008).^2^

In this cross-sectional, descriptive study, we reported descriptive statistics, including mean age and incidence of cytogenetic abnormalities, using the SPSS software package (version 18). Moreover, we performed comparisons in terms of cytogenetic subclasses and age groups using the Pearson chi-square test with MED CALC software.

## Results

We conducted a cytogenetic analysis of 168 ALL patients, comprising 154 B-ALL and 14 T-ALL cases. 

The 154 B-ALL patients were comprised of 53 females at a mean age of 12.13 ± 14.07 years and 101 males at a mean age of 14.65±15.76 years (mean age=13.78±15.2 years, range=1 month to 79 years). Children accounted for 108 (70.1%) cases at a mean age of 5.79±3.73 years (lower than 15 years), and adults comprised 46 (29.9%) cases at a mean age of 35.36±14.82 years. 

The 14 T-ALL patients were composed of 5 (35.7%) children and 9 (64.2%) adults, and all of them, with the exception of one, were male (92.9%). 

**Figure 1:**
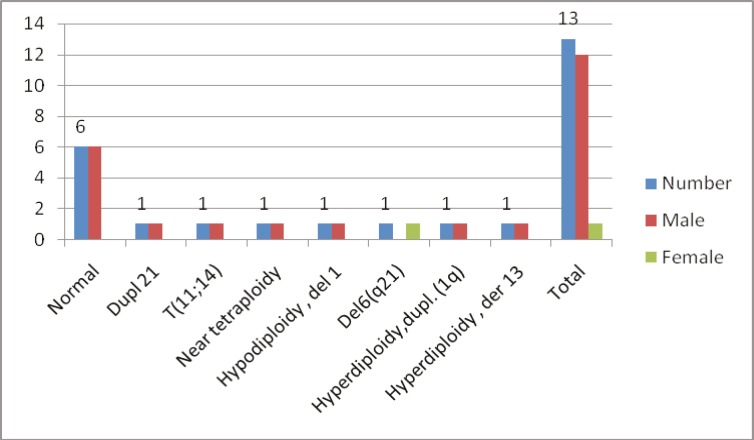
This graph illustrates the distribution of cytogenetic abnormalities in our T-cell acute lymphoblastic leukemia patients.

**Figure 2:**
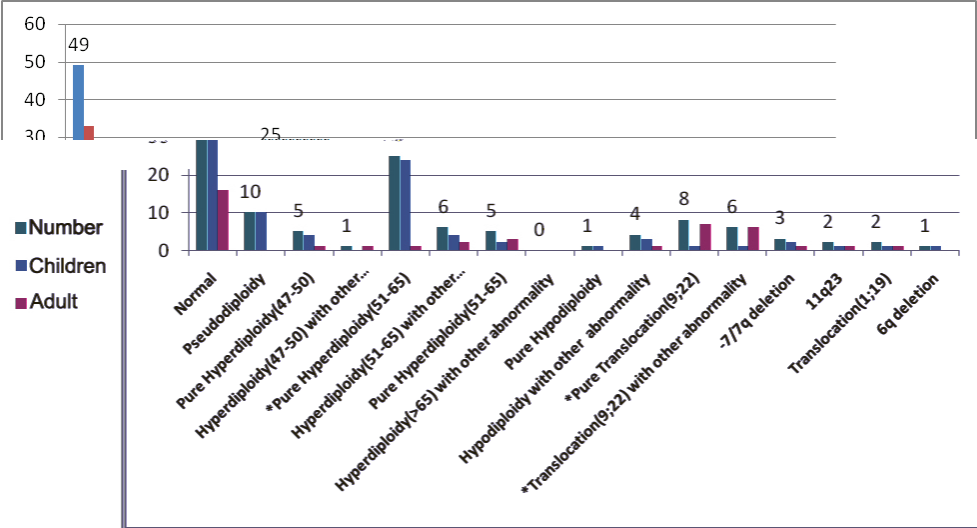
This graph depicts the distribution of the karyotypes of our 128 B-precursor acute lymphoblastic leukemia patients. *P value is statistically significant

Karyotyping was unsuccessful in 26 patients: 15 specimens were cultured but did not have metaphases and 11 samples had too few metaphases to be adequate or had too poor quality to be interpreted. There were 128 cases of successful cytogenetic analysis of B-ALL patients, with 49 (38.3%) cases, 16 (12.5%) adults and 33 (25.7%) children, showing normal karyotypes. Normal karyotypes were found in 6 out of the 14 (46.1%) T-ALL patients. The frequency of cytogenetic abnormalities, including numerical and/or structural changes, was 61.7% and 53.8% in the B-ALL and T-ALL patients, respectively. There were 13 T-ALL patients with successful karyotyping: 6 (46.15%) patients had normal karyotype and the main abnormalities were Dup21, del 6q21, der 13, dup 1, t(11;14), near tetraploidy, and del 1. Figure 1 and table 1 depict the distribution of the cytogenetic abnormalities in the T-ALL patients. The main cytogenetic abnormality was hyperdiploidy (47 to >65 chromosomes) in 42 (32.8%) B-ALL patients. In the children group, the most common abnormality was hyperdiploidy in 34 (38.6%) patients in comparison with the adults, in whom hyperdiploidy was found in 8 (20%) patients. Hyperdiploidy with 51-65 chromosomes, as the sole abnormality, was significantly more frequent in the children (24/27.3%) than in the adults (1/2.5%) (P<0.05). Deletions, duplications, and translocations were the main structural chromosomal abnormalities. In the pseudodiploid group, the most common abnormal karyotype was t(9;22) in 14 patients. This group included 12 (30%) adults and 2 (2.2%) children. Other abnormalities in the pseudodiploid group were t(1;19) in 2 (1.5%) patients, -7/7q deletion in 3 (2.3%), 11q23 abnormality in 2 (1.5%), and 6q del in one (0.8%) (figure 2 and table 2). Other pseudodiploidy karyotype groups (except for the main abnormalities) were detected in the second large group of cytogenetic abnormalities in 10 (7.8%) patients. These abnormalities were t(10;12), inv12, t(4;9), t(1;4), t(7;14), del X, dup 1, and del 12 (each of them in one [1.13%] patient and t(6;12) in 2 [2.27%] patients). The details of these abnormalities are provided in table 3.

**Table 1:** Distribution of the cytogenetic abnormalities in the T-cell acute lymphoblastic leukemia patients

**Karyotype**	**Frequency %**	**Male/Female**
Normal	6(46.15)	6/0
Dupl 21	1(7.69)	1/0
t(11;14)	1(7.69)	1/0
Near tetraploidy	1(7.69)	1/0
Hypodiploidy , del 1	1(7.69)	1/0
Del6(q21)	1(7.69)	0/1
Hyperdiploidy, dupl. (1q)	1(7.69)	1/0
Hyperdiploidy , der 13	1(7.69)	1/0
Total	13(100)	12/1

**Table 2:** Distribution of the karyotypes of 128 B-precursor acute lymphoblastic leukemia patients

**Group**	**Frequency/%**	**Children**	**Adult**	**P value**
Normal	49/38.3%	33/37.5%	16/40%	0.94
Pseudodiploidy	10/7.8%	10/11.4%	0	0.41
Hyperdiploidy (47-50):				0.96
A) pure	5/3.9%	4/4.5%	1/2.5%	
B) other abnormalities	1/0.8%	0	1/2.5%	
Hyperdiploidy (51-65):				
A) pure	25/19.5%	24/27.3%	1/2.5%	0.0024
B) other abnormalities	6/4.7%	4/4.5%	2/5%	0.74
Hyperdiploidy (>65):				0.36
A) pure	5/3.5%	2/2.3%	3/7.5%	
B) other abnormalities	0	0	0	
Hypodiploidy:				0.78
A) pure	1/0.8%	1/0.8%	0	
B) other abnormalities	4/3.1%	3/3.4%	1/2.5%	
Translocation (9;22):				
A) pure	8/6.3%	1/1.1%	7/17.5%	0.0009
B) other abnormalities	6/4.7%	1/1.1%	5/12.5%	0.0017
-7/7q deletion	3/2.3%	2/2.3%	1/2.5%	0.57
11q23	2/1.5%	1/1.1%	1/2.5%	
Translocation (1;19)	2/1.5%	1/1.1%	1/2.5%	
6q deletion	1/0.8%	1/1.1%	0	
Total	128/100%	88/100%	40/100%	

**Table 3:** Distribution of the other cytogenetic abnormalities in pseudodiploid B-precursor acute lymphoblastic leukemia pediatric patients

**Abnormalities**	**Number of cases **	**Percentages of cases ** **(in total 88 children)**
t(10;12), inv12	1	1.13
t(4;9)	1	1.13
t(1,4)	1	1.13
t(7;14)	1	1.13
t(6;12)	2	2.27
del X	1	1.13
dup 1	1	1.13
del 12p	2	2.27
Total	10	11.4

## Discussion

In this study, we present cytogenetic findings on 168 adult and pediatric ALL patients in Fars province and compare our findings with the relevant reports in the literature. We had a successful cell culture rate of 84.5%, which is comparable to those in the studies by Silva et al.^8^ and Pérez-Vera et al.^5^ who had successful cell culture rates of 91% and 87.5%, respectively. Unsuccessful cell cultures may be due to the nature of malignant cells as well as technical and transport problems.

In the present study, abnormal karyotypes were found in 61.7% of our B-ALL cases. Usvasalo et al.^9^ showed that using advanced methods in cytogenetics such as polymerase chain reaction (PCR) and fluorescent *in situ* hybridization (FISH) could augment the detection of abnormal cytogenetics in leukemic patients and, the authors detected chromosomal aberration in 85% of the cases. Silva et al.^8^ detected abnormal cytogenetics in 92.3% of their patients. These differences are due to the use of the conventional G-banding technique versus more developed cytogenetic analytical methods. In the present study, 38.3% of our B-ALL cases showed normal karyotypes, which was similar to the figure reported by the Xin Li et al.^4^ study (39%). There were slightly fewer normal karyotypes in our pediatric B-ALL patients (37.5%) than in our adult B-ALL patients (40%); however, the difference was not statistically significant (P>0.05). In the B-ALL group, t(9;22) was the most frequent chromosomal translocation (11%). Moorman et al.^10^ reported 19% positivity of this translocation, but they performed both FISH and RT-PCR. Mancini et al.^11^ reported 388 ALL cases with a median age of 31 years with 16.9% Philadelphia chromosomes. The median age of our group was 13.78±15.20 years, so the difference may be explained by the fact that our results came from a different age group.

In our study, 32.8% of all the B-ALL patients showed hyperdiploidy(47 to >65 chromosomes) (Note that 27.3% of them showed only numerical changes.) Pure hyperdiploidy with 51-65 chromosomes had a significantly higher incidence in the children than in the adults (27.3 vs. 2.5%) (P<0.005). Xin Li et al.^4^ reported hyperdiploidy of more than 50 chromosomes in 17.5% of their children and 3.4% of their adults; the results were similar to those reported elsewhere in the literature (20-40% vs. 2-15%). Hyperdiploidy is one of the most frequent abnormalities which are found in up to 25% of adult ALL cases. Hyperdiploid patients with a chromosomal number of more than 50 have a better prognosis; this is more frequent in children than in adults.^12^ We reported 5 (3.9%) B-ALL cases who had 45 chromosomes and one T-ALL patient who had 41 chromosomes. In the literature, hypodiploid chromosome numbers were found in 3-10% of adults and 1-7% of childhood ALL.^13^ Xin Li et al.^4^ found that 4.9% of children and 4% of adults showed hypodiploidy with 40-45 chromosomes. In our study, most of the hypodiploid cases were children (4 [4.2%]). Thus, our results are in the same range as those reported previosuly.^8^^,^^13^

We did not find translocation (12;21) in our study population, but we found 2 cases with 12p deletion as the sole abnormality. In the pseudodiploidy group, cryptic translocation of (12; 21) was the most common abnormality (21%) in the Xin Li et al.^4^ study in children. The authors, however, detected this abnormality by the RT-PCR technique. This abnormality is difficult to detect and needs advanced molecular techniques such as the Southern blot or RT-PCR and FISH analysis.^12^ Translocation (12;21) has been reported to be the most common molecular-karyotypic abnormality detected in pediatric leukemic patients and may be particularly associated with late relapse.^12^ We did not find this translocation among our study population; accordingly, for leukemia patients without specific translocations, we recommend the use of other methods such as FISH or RT-PCR as complementary techniques. We found t(1;19) in 2 (1.2%) cases, but this translocation has been reported in 3-6% of cases elsewhere in the literature.^14^ This difference might be due to the limited number of cases in our study.

We found a low incidence of other abnormalities, including 11q23 in 1.5% of the study patients, by comparison with the figure in the other published studies in the pediatric age group (2%).^12^^,^^15^ Rarity of this aberration in our group may be owing to the fact that the occurrence of this abnormality is more frequently seen in infants under one year and in secondary ALL.^5^

Translocations such as t(7;14), t(10;12), t(4;9), and t(7;9) were found in some cases in our study, but we did not find any similar report in the literature. We also found chromosomal structural changes such as deletion 7, deletion 6q, deletion X, duplication 1, and deletion 12p (table 3). Some of these changes such as the deletion or loss of chromosome 7 are more frequently seen in acute myeloid leukemia (AML) and myelodysplastic syndrome (MDS) patients. We found no report on inversion 16 , t(1;4), and t(10;13) in ALL patients in the literature, but t(7;14), t(7;9), and t(6,12) were reported in T-ALL cases.^16^^,^^17^ Translocation (4;9) has also been previously reported in AML patients.^18^

Given the limited number of cases in the present study, the type and frequency of some the abnormalities are different from those reported by other groups. 

## Conclusion

Cytogenetic analysis in ALL plays an important role in the classification and prognosis of the patients. The present study was the first of its kind to survey the distribution of cytogenetic abnormalities in pediatric and adult ALL patients in Fars Province. In comparison to the other relevant studies, we found that normal karyotypes in our study population were more frequent than those in the other studies and that the difference between the children and adults did not constitute statistical significance. Hyperdiploidy was the most frequent abnormal karyotype in our study, which chimes in with the literature. Pure hyperdiploidy had a significantly higher incidence in children than in adults. The frequencies of some other chromosomal aberrations such as t(9;22) were comparable to those reported elsewhere. Other abnormalities, including 11q23 and t(1;19), had low incidence rates compared to the figures reported previously. Finally, we found abnormalities such as the deletion or loss of chromosome 7, which are more frequently reported in AML or MDS patients. We conclude that advanced molecular methods which can detect cryptic abnormalities in ALL cases must be utilized routinely in cytogenetic laboratories. We also recommend that the prognostic effect of cytogenetic abnormalities for ALL patients be evaluated in the future. 

## References

[1] Dastgiri S, Fozounkhah Sh, Shokrgozar S, Taghavinia M, Asvadi A (2011). Incidence of Leukemia in the Northwest of Iran. Health Promotion Perspectives.

[2] Hutchison RE, Schexneider KI, McPherson RA, Pincus MR, editors (2011). Leukocyte disorder. Henrey’s clinical diagnosis and management by laboratory methods.

[3] King RL, Naghashpour M, Watt CD, Morrissette JJ, Bagg A (2011). A comparative analysis of molecular genetic and conventional cytogenetic detection of diagnostically important translocations in more than 400 cases of acute leukemia, highlighting the frequency of false-negative conventional cytogenetics. Am J Clin Pathol.

[4] Li X, Li J, Hu Y, Xie W, Du W, Liu W (2012). A comprehensive cytogenetic classification of 1466 Chinese patients with de novo acute lymphoblastic leukemia. Leuk Res.

[5] Pérez-Vera P, Mújica-Sánchez M, Carnevale A, Rivera-Luna R, Paredes R, Martínez A (2001). Cytogenetics in acute lymphoblastic leukemia in Mexican children: an institutional experience. Arch Med Res.

[6] Behjati F, Akbari MT, Ghavamzadeh A, Izadyar M (2001). Chromosomal abnormalities in leukemia in Iran: a pilot study. Archives of Iranian Medicine.

[7] Shaffer LG, Slovak ML Campbell LJ (2009). An international system for human cytoenetic nomenclature.

[8] Silva ML, Ornellas de Souza MH, Ribeiro RC, Land MG, Boulhosa de Azevedo AM, Vasconcelos F (2002). Cytogenetic analysis of 100 consecutive newly diagnosed cases of acute lymphoblastic leukemia in Rio de Janeiro. Cancer Genet Cytogenet.

[9] Usvasalo A, Räty R, Harila-Saari A, Koistinen P, Savolainen ER, Vettenranta K (2009). Acute lymphoblastic leukemias with normal karyotypes are not without genomic aberrations. Cancer Genet Cytogenet.

[10] Moorman AV, Harrison CJ, Buck GA, Richards SM, Secker-Walker LM, Martineau M (2007). Karyotype is an independent prognostic factor in adult acute lymphoblastic leukemia (ALL): analysis of cytogenetic data from patients treated on the Medical Research Council (MRC) UKALLXII/Eastern Cooperative Oncology Group (ECOG) 2993 trial. Blood.

[11] Mancini M, Scappaticci D, Cimino G, Nanni M, Derme V, Elia L (2005). A comprehensive genetic classification of adult acute lymphoblastic leukemia (ALL): analysis of the GIMEMA 0496 protocol. Blood.

[12] Jabber Al-Obaidi MS, Martineau M, Bennett CF, Franklin IM, Goldstone AH, Harewood L (2002). ETV6/AML1 fusion by FISH in adult acute lymphoblastic leukemia. Leukemia.

[13] Onciu M (2009). Acute lymphoblastic leukemia. Hematol Oncol Clin North Am.

[14] Paulsson K, Horvat A, Fioretos T, Mitelman F, Johansson B (2005). Formation of der(19)t(1;19)(q23;p13) in acute lymphoblastic leukemia. Genes Chromosomes Cancer.

[15] Mann G, Cazzaniga G, van der Velden VH, Flohr T, Csinady E, Paganin M (2007). Acute lymphoblastic leukemia with t(4;11) in children 1 year and older: The ‘big sister’ of the infant disease. Leukemia.

[16] Soulier J, Clappier E, Cayuela JM, Regnault A, García-Peydró M, Dombret H (2005). HOXA genes are included in genetic and biologic networks defining human acute T-cell leukemia (T-ALL). Blood.

[17] Speleman F, Cauwelier B, Dastugue N, Cools J, Verhasselt B, Poppe B (2005). A new recurrent inversion, inv(7)(p15q34), leads to transcriptional activation of HOXA10 and HOXA11 in a subset of T-cell acute lymphoblastic leukemias. Leukemia.

[18] Grand FH, Waghorn K, Ernst T, Ohyashiki K, Cross NC (2011). The t(4;9)(q11;q33) fuses CEP110 to KIT in a case of acute myeloid leukemia. Leukemia.

